# Prevalence and Clinical Significance of Occult Pulmonary Infection in Elderly Patients with Type 2 Diabetes Mellitus

**DOI:** 10.1155/2021/3187388

**Published:** 2021-12-02

**Authors:** Jian Hua, Ping Huang, Honghui Liao, Xiaobing Lai, Xiaoyi Zheng

**Affiliations:** ^1^Zhuantang Community Healthcare Center, Hangzhou 310024, China; ^2^The Second Affiliated Hospital of Zhejiang Chinese Medical University, Hangzhou 310005, China

## Abstract

The occult pulmonary infection is the most common complications in elderly patients with type 2 diabetes mellitus (T2DM). Since its etiological characteristics has not been clarified, infection control remains a serious problem for public health. To investigate the prevalence and clinical significance of occult pulmonary infection in elderly T2DM patients, in this study, 573 elderly patients cochallenged with T2DM and community-acquired pulmonary infection from January 2018 to December 2020 were selected in the hospitals and divided into occult pneumonia group (OP, *n* = 249) and nonoccult pneumonia group (NOP, *n* = 324) according to the nature of infection. Clinical medical records were analyzed retrospectively to summarize the infection characteristics of elderly diabetics with occult pneumonia. The prevalence of the cases (278/324, 85.8%) in NOP group was not higher than that in OP group (206/249, 82.7%; *P* > 0.05). Also, there was not significant difference in the distribution of isolated pathogens among the positive patients. The length of hospitalization and mortality of OP patients were significantly higher than those NOP patients. Multivariate logistic regression showed that advanced age, comorbidities, hypothyroidism, senile dementia, and prolonged bed rest were independent risk factors for occult pneumonia in elderly diabetic patients. Therefore, the results demonstrated that the pulmonary infection in elderly patients with diabetes mellitus is often occult. Gram-negative bacteria are the predominant pathogens and cause poor prognosis. Advanced age, comorbidities (senile dementia, hypothyroidism), and prolonged bed rest are the independent risk factors for occult pneumonia.

## 1. Introduction

Diabetes mellitus is an endocrine and metabolic disease with high incidence and genetic tendency, which seriously affects the quality of life of patients and their offspring [1]. In the 1980s, the incidence of diabetes mellitus in China was only 1%. Nowadays, diabetics in China account for about 1/5 of the world's total. More than 90% of the diabetic patients in China suffer from type 2 diabetes mellitus (T2DM), and the incidence is increasing year by year [2]. Early onset of T2DM is hidden and hard to cure after onset, which brings heavy financial burden to families and society [3–5]. At present, the specific pathogenesis of T2DM is not clear. It is generally believed to be caused by the interaction of various factors such as obesity, abnormal glucose and lipid metabolism, and inflammatory reactions [6–9].

Clinical trials showed that the incidence of pulmonary infection in patients with T2DM was significantly higher than that in nondiabetic patients, and the older the patient is, the greater the vascular fragility, with poor blood glucose control, deterioration of immune function, and a higher risk of pulmonary infections [10–12]. Occult pulmonary infection, which is more common in elderly patients, is a kind of pulmonary infection with no obvious respiratory symptoms [13–15]. It is difficult to determine the location of lesion and make initial diagnosis. At present, scholars do not have a precise definition and diagnostic criteria for occult pulmonary infection, but most elderly patients have multiple underlying diseases. Pulmonary infection with atypical clinical symptoms may lead to missed diagnosis, misdiagnosis, neglect of the disease, and missing the optimal opportunity for treatment, which may result in malignant progress of the disease and even endanger lives of patients [16]. Thus, it should be paid more attention. At present, the research on occult pulmonary infection is still in its infancy at home and abroad. Understanding the clinical status and risk factors of occult pulmonary infection in elderly patients with T2DM is of great clinical significance for controlling infection and improving the prognosis of patients. In this study, the case data of elderly patients with T2DM complicated with community-acquired pulmonary infection were retrospectively analyzed; the clinical etiological characteristics, treatment, and prognosis of such patients were summarized; and their risk factors were preliminarily analyzed to provide reference for clinical intervention.

## 2. Method

### 2.1. Study Population and Definitions

**Figure 1:**
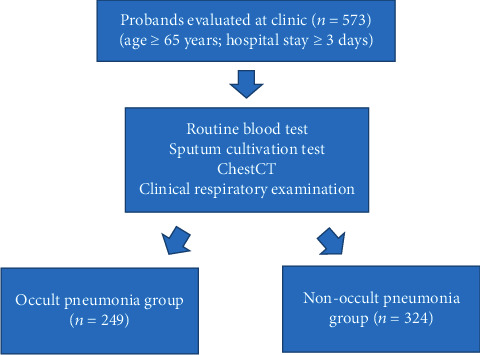
The patients underwent a comprehensive evaluation, including routine blood test, sputum cultivation test, chest CT, and clinical respiratory examination.

Totally, 573 elderly patients with T2DM complicated with community-acquired pulmonary infection treated in the second affiliated hospital of Zhejiang Chinese Medical University from January 2018 to December 2020 were selected. Inclusion criteria were employed as follows: (1) meeting diagnostic criteria of T2DM [17]; (2) meeting diagnostic criteria for community-acquired pulmonary infection [18]; (3) age ≥ 65 years; (4) hospital stay ≥3 days; and (5) clinical data fully available. Exclusion criteria: (1) complicated with infection of other sites; (2) changes in leukocyte level caused by other noninfectious diseases; (3) presence of immune system disorders; (4) antimicrobial drugs taken before the first collection of pathogenic specimens; (5) complicated with other chronic pulmonary diseases. The patients were divided into occult pneumonia group and nonoccult pneumonia group according to whether the pulmonary infection is occult or not. Diagnostic criteria for occult pneumonia were employed as follows: (1) no typical respiratory symptoms, including cough, sputum, and chest pain; (2) diagnosis confirmed by computed tomography (CT); (3) patients with no obvious abnormalities in CT and unable to complete CT examination, positive sputum culture or blood culture, and elevated inflammatory indicators are effective after empirical anti-infective therapy. Also, the indexes of blood routine, urine routine, and hepatic and renal function were examined to exclude vasculitis, and the throat swabs were detected by RT-PCR analysis to exclude various acute infection including COVID-19. The flow chart of the study protocol and diagnostic work up was presented in Figure 1. This study has been approved by the ethics committee of the second affiliated hospital of Zhejiang Chinese Medical University.

### 2.2. Clinical Information

Clinical data of patients were collected, including age, gender, living habits (smoking history, drinking history), underlying diseases (hypertension, coronary disease, senile dementia, etc.), hypothyroidism, invasive operations (invasive airway operation and indwelling catheter), nasal feeding, long-term bed rest, glucocorticoid use, results of etiology and drug sensitivity analysis, length of hospital stay, and mortality. The biochemical indicators, such as white blood cell (WBC), procalcitonin (PCT), and albumin (ALB), were detected by automatic biochemical analyzer (HITACHI 7180, Japan) in the serum. Sputum samples were collected from the patients at the second affiliated hospital of Zhejiang Chinese Medical University and cultured in blood agar media for the microbiological and genomic analysis. Positive cultures with colonies suspected of lethal pathogens and resistant bacteria were sent to the laboratory of microbiology of hospital for further DNA extraction.

### 2.3. Statistical Analysis

GraphPad Prism 9 (GraphPad Software, San Diego, USA) was used for data processing and analysis. Measurement data were presented as mean ± standard deviation. Comparisons were by *T*-test. Enumeration data were presented in rate (%). Comparisons were made by *χ*^2^-test. The risk factors of occult pneumonia in elderly diabetic patients were analyzed by logistic regression. *P* < 0.05 was taken as statistically significant.

## 3. Result

### 3.1. Clinical Characteristics

After a detailed assessment according to the inclusion and exclusion criteria, a total of 573 subjects were considered in the analysis. Their mean age was 72.7 ± 8.8 years, and male patients were 53.6%. A history of smoking was present in 33.0% and drinking in 14.5%. All the patients had basic diseases, in which 16.8% had two more basic diseases.

### 3.2. Clinical Characteristics of Pulmonary Infection in T2DM Patients

The distribution of pathogenic bacteria detected in the two groups was shown in Table 1. Totally, 206 induced sputum samples were positive to the pathogenic bacteria culture in 249 patients of the occult group (OP), whose positive rate was 82.7%, and 157 kinds of pathogenic bacteria were detected among 249 OP samples. In other hand, 278 samples were positive in 324 patients of the nonoccult group (NOP), whose positive rate was 85.8%, and 183 kinds of pathogenic bacteria were detected. However, there was no significantly difference in the positive rate of pathogen culture between the two groups (*χ*^2^ = 1.012, *P* > 0.05). Although the components of pathogens detected in positive patients were not identical, there was no significantly difference in the distribution of pathogens detected in positive patients (*χ*^2^ = 2.519, *P* > 0.05).

### 3.3. Treatment and Prognosis of Pulmonary Infection in T2DM Patients

All patients received conventional treatments, such as blood glucose reduction, anti-infection, and nutritional replenishment. The empirical anti-infection treatment was employed by mainly using antimicrobial drugs such as carbapenems and *β* lactam/enzyme inhibitors. When the condition did not improve, sensitive drugs were selected according to the results of drug sensitivity. Imipenem and vancomycin were used cautiously in patients with resistance changes. In the contrast of the prognosis of the two groups as shown in Table 2, patients in the occult group had a higher length of hospital stay and case fatality rate than those in the nonoccult group (*P* < 0.05).

### 3.4. Univariate Analysis of Occult Pneumonia in T2DM Patients

As shown in Table 3, the results by using univariate analysis presented that there were significantly differences in age, senile dementia, hypothyroidism, WBC, PCT, C-reactive protein (CRP), and prolonged bed rest between the two groups (*P* < 0.05). It indicated that those indexes could contribute to the development and outcome of the occult pneumonia in T2DM patients.

### 3.5. Multivariate Analysis of Occult Pneumonia in T2DM Patients

As showed in Table 4, the results obtained from multivariate logistic regression analysis presented that advanced age, senile dementia, hypothyroidism, and prolonged bed rest were independent risk factors for occult pneumonia in elderly diabetic patients (*P* < 0.05), whose OR values were all more than 1, and *P* values were less than 0.05, indicating the statistical significance.

## 4. Discussion

Numerous studies have confirmed that diabetes is an important risk factor for pulmonary infection. Several reasons have been found: (1) high blood glucose levels provide good growth conditions for pathogenic bacteria, which facilitates their mass multiplication; (2) hyperglycemia increases plasma osmotic pressure and attenuates the chemotaxis, phagocytosis, and bactericidal ability of neutrophils, resulting in decreased clearance of pulmonary pathogens; (3) hyperglycemia can reduce cellular immunity and the body's anti-infective ability, increasing the risk of infection; (4) diabetes mellitus is often associated with uremia, ketoacidosis, and other complications. The patients have metabolic disorders, negative nitrogen balance, and a higher probability of infection. And in patients with vasculopathy, reduced tissue blood flow also reduces the absorption of antibiotics [19–22]. Occult pneumonia refers to insidious clinical symptoms and/or insidious lesion sites, which are not easily diagnosed at the initial diagnosis, often without typical symptoms of respiratory tract infection such as cough, sputum, and chest pain, and are mostly seen in the elderly population. At present, there are few clinical studies on occult pneumonia, and there are no accepted definitions and diagnostic criteria. According to the previous reports, elderly patients were the high-risk population of occult pulmonary infection. Elderly patients have a substantially increased risk of pulmonary infection since all physiological functions of the body were in an attenuated state, and their typical respiratory symptoms were very easily masked by underlying diseases. When the pulmonary infection overlaps with the sagittal position of the heart or spine, chest X-ray (CXR) often fails to confirm the diagnosis, resulting in misdiagnosis or delay in anti-infective treatment and even multiorgan dysfunction, thus endangering patient's life [11, 23–25]. Therefore, understanding the clinical manifestations and risk factors of occult pulmonary infections in elderly diabetic patients is important for controlling infections and improving prognosis.

In this study, the predominant pathogens of elderly patients with occult pulmonary infection and nonoccult pulmonary infection were all Gram-negative bacteria, marked by *Pseudomonas aeruginosa*, *Acinetobacter baumannii*, and *Klebsiella pneumoniae*. The results were basically consistent with previous reports, and the inconsistency in specific proportions might be related to different geographical environments [12, 26]. Moreover, patients in both groups were treated with comprehensive treatment, and the treatment was basically similar. After the diagnosis of pulmonary infection in elderly patients, it is necessary to carefully assess the severity of the disease with reference to the physiological characteristics of the elderly population and select sensitive antibiotics to control the infection. Prompt drug replacement is necessary in case of resistance changes in the causative organism. For patients who are receiving many antibiotics and invasive procedures, the possibility of drug-resistant strains should be considered. Patients with long-term medication and malnutrition should be cautioned against the occurrence of superinfection. In addition, patients in the occult group had longer hospital stays and a higher case fatality rate, suggesting that the prognosis of elderly patients with diabetes mellitus complicated with occult pulmonary infection was worse.

It has been known that the patients with T2DM are prone to pneumonia for a variety of reasons, including impairment of immune function, impairment of pulmonary function, and ischemia-hypoxia due to hyperglycemia-induced collage synthesis decrease and secondary vascular endothelial changes [11]. In elderly patients with severe pneumonia, fasting glucose values ≥11 mmol/L and glycosylated hemoglobin >7% were significantly and positively associated with increased risk of death. High glycosylated hemoglobin was an independent risk factor for increased mortality risk [12, 27]. However, oral antidiabetic drugs have been demonstrated to be associated with community-acquired pneumonia [28]. Notably, any combination with thiazolidinediones and other antidiabetic drugs had been considered to increase higher risk of community-acquired pneumonia, while the use of DPP-4 inhibitors or metformin dose not display this danger [29–32].

We also found that the risk factors of occult pulmonary infection in elderly patients with T2DM were analyzed. The results showed that age, senile dementia, hypothyroidism, WBC, PCT, ALB, and prolonged bed rest were related to occult pulmonary infection, and that advanced age, senile dementia, hypothyroidism, and prolonged bed rest were independent risk factors for occult pneumonia. Patients of advanced age have a low capacity to respond to stress. This was because mass neutrophils remain in the viscera and the adhesion of neutrophils was enhanced after infection. Their peripheral blood levels were not increased but decreased. The body temperature might also decrease or remain normal. More cough reflex inhibition made the cough symptoms mild or without cough. The onset was insidious due to lack of fever, cough, and other typical clinical symptoms [33]. For patients with senile dementia, the brainstem had poor regulation of respiratory function, and the lungs had a greatly diminished ability to clear pathogens, which, combined with the fact that they often present with language impairment and had difficulty expressing subjective sensation, thereby delaying the treatment [34]. Studies have shown that multiple neuroendocrine hormone abnormalities are present in patients with diabetes mellitus, manifested by glucocorticoid hypersecretion and dysfunction of hypothalamo-hypophyseal-thyroidal axis (HHTA) [35–37]. Moreover, with increasing age, HHTA also becomes progressively aged, leading to hypothyroidism manifested by reduced respiration, hypothermia, and hyporeflexia, which can mask infectious symptoms and lead to occult pulmonary infections [38–40]. In addition, long-term bed ridden patients have relatively elevated diaphragmatic position, and the scope of their imaging ghosting is enlarged, making it difficult to obtain good and comprehensive evidence from chest X-ray imaging. Therefore, the infection could be easily concealed.

In summary, the pulmonary infections in elderly patients with diabetes mellitus are often occult. The predominant pathogens are Gram-negative bacteria. The prognosis is poor. Advanced age, comorbidities (senile dementia, hypothyroidism), and prolonged bed rest are the independent risk factors for occult pneumonia. Clinical attention should be paid to the following points for such elderly patients: (1) improve lung CT examination and laboratory inflammatory indicator test as soon as possible to raise the detection rate of occult pulmonary infection; (2) aggressively give anti-infective treatment after the diagnosis of occult pulmonary infection; select sensitive antibiotics and adjust medication regimen in a timely manner according to treatment effect; (3) aggressively treat the underlying diseases of elderly patients, especially those with senile dementia and hypothyroidism, to avoid occult pulmonary infections. However, this study is a retrospective study with a low level of evidence. Future efforts will be made to carry out prospective, multicenter, and large-sample studies to further analyze the status of occult pulmonary infections in elderly patients with endocrine diseases.

## Figures and Tables

**Table 1:** The distribution of pathogenic bacteria detected from the patients in OP and NOP groups.

Pathogens	OP (*n* = 249)	NOP (*n* = 324)
Gram-negative bacteria	**134 (53.8%)**	**198 (61.1%)**
*Klebsiella pneumoniae*	34 (13.7%)	69 (21.3%)
*Pseudomonas aeruginosa*	39 (15.7%)	55 (17.0%)
*Acinetobacter baumannii*	36 (14.5%)	28 (8.6%)
*Escherichia coli*	9 (3.6%)	21 (6.5%)
*Enterobacter cloacae*	0 (0.0%)	18 (5.6%)
*Pseudomonas maltophilia*	16 (6.4%)	7 (2.2%)
Gram-positive bacteria	**79 (31.7%)**	**80 (24.7%)**
*Streptococcus pneumoniae*	30 (12.0%)	23 (7.1%)
*Staphylococcus aureus*	22 (8.8%)	30 (9.3%)
*Staphylococcus epidermidis*	18 (7.2%)	16 (4.9%)
*Hemolytic Staphylococcus*	9 (3.6%)	0 (0.0%)
*Enterococcus*	0 (0.0%)	11 (3.4%)
Fungi	**36 (14.5%)**	**46 (14.2%)**
*Candida albicans*	19 (7.6%)	33 (10.2%)
*Candida tropicalis*	12 (4.8%)	11 (3.4%)
Others	5 (2.0%)	2 (0.6%)

**Table 2:** The prognostic indicators between the patients in OP and NOP groups.

Prognostic indicators	OP (*n* = 249)	NOP (*n* = 324)	*P* value
Length of hospitalization (d)	18.5 ± 4.8	11.6 ± 3.3	*P* < 0.01
Mortality (%)	20 (8.0%)	9 (2.78%)	P <0.01

**Table 3:** Univariate analysis of the occult pneumonia in the patients with T2DM.

Clinicopathologic characteristics		OP (*n* = 249)	NOP (*n* = 324)	*t* value	*P* value
Age (years)	65-70	119	164	21.02	<0.01
	71-80	76	132		
	>80	54	28		

Gender	Male	132	175	0.06	0.81
	Female	117	149		

History of smoking	Smoked	89	100	1.52	0.22
	Never smoked	160	224		

History of drinking	Drank	44	39	3.61	0.06
	Never drank	205	285		

Underlying diseases	Hypertension	95	126	0.03	0.86
	Coronary heart disease	59	74	0.06	0.81
	Senile dementia	83	51	24.32	<0.01
	Hypothyroidism	72	61	8.04	<0.01

Biochemical indexes	WBC (×10^9^/mL)	8.65 ± 1.62	10.87 ± 2.67	11.58	<0.01
	PCT (*μ*g/L)	0.18 ± 0.02	0.21 ± 0.02	17.80	<0.01
	CRP (mg/L)	34.6 ± 8.6	32.4 ± 5.6	3.70	<0.01
	ALB (g/L)	33.7 ± 5.9	34.5 ± 6.4	1.53	0.13

Invasive operation	Have	54	75	0.17	0.68
	Not	195	249		

Prolonged bed rest	Prolonged	45	66	0.48	0.49
	Never prolonged	204	258		

**Table 4:** Multivariate analysis of the occult pneumonia in the patients with T2DM.

Characteristics	*β*	SE	Wald value	OR value	95% CI	*P* value
Age	0.69	0.32	4.67	1.98	1.07-3.69	0.03
Senile dementia	0.39	0.17	5.25	1.47	1.06-2.04	0.02
Hypothyroidism	0.41	0.20	4.53	1.51	1.03-2.21	0.03
Prolonged bed rest	0.24	0.09	6.64	1.27	1.06-1.52	0.01

SE: standard error; OR: odds ratio; CI: confidence interval.

## Data Availability

The data used to support the findings of this study are included within the article.
